# Organizational interventions employing principles of complexity science have improved outcomes for patients with Type II diabetes

**DOI:** 10.1186/1748-5908-2-28

**Published:** 2007-08-28

**Authors:** Luci K Leykum, Jacqueline Pugh, Valerie Lawrence, Michael Parchman, Polly H Noël, John Cornell, Reuben R McDaniel

**Affiliations:** 1 South Texas Veterans Health Care System, University of Texas Health Science Center at San Antonio, San Antonio TX, 78229, USA; 2 Department of Family and Community Medicine, University of Texas Health Science Center at San Antonio, San Antonio TX, 78229, USA; 3 McComb's School of Business, University of Texas at Austin, Austin TX, USA

## Abstract

**Background:**

Despite the development of several models of care delivery for patients with chronic illness, consistent improvements in outcomes have not been achieved. These inconsistent results may be less related to the content of the models themselves, but to their underlying conceptualization of clinical settings as linear, predictable systems. The science of complex adaptive systems (CAS), suggests that clinical settings are non-linear, and increasingly has been used as a framework for describing and understanding clinical systems. The purpose of this study is to broaden the conceptualization by examining the relationship between interventions that leverage CAS characteristics in intervention design and implementation, and effectiveness of reported outcomes for patients with Type II diabetes.

**Methods:**

We conducted a systematic review of the literature on organizational interventions to improve care of Type II diabetes. For each study we recorded measured process and clinical outcomes of diabetic patients. Two independent reviewers gave each study a score that reflected whether organizational interventions reflected one or more characteristics of a complex adaptive system. The effectiveness of the intervention was assessed by standardizing the scoring of the results of each study as 0 (no effect), 0.5 (mixed effect), or 1.0 (effective).

**Results:**

Out of 157 potentially eligible studies, 32 met our eligibility criteria. Most studies were felt to utilize at least one CAS characteristic in their intervention designs, and ninety-one percent were scored as either "mixed effect" or "effective." The number of CAS characteristics present in each intervention was associated with effectiveness (p = 0.002). Two individual CAS characteristics were associated with effectiveness: interconnections between participants and co-evolution.

**Conclusion:**

The significant association between CAS characteristics and effectiveness of reported outcomes for patients with Type II diabetes suggests that complexity science may provide an effective framework for designing and implementing interventions that lead to improved patient outcomes.

## Background

Although the cost of managing patients with chronic disease is high, and is predicted to rise to 80% of US health care dollars by 2020 [1], identification of effective ways to improve care of patients with chronic disease has been difficult. While the evidence base regarding optimal medical management of these patients has grown, our ability to implement evidence into routine clinical practice across diverse clinical settings has been limited. A recent study of chronically ill patients revealed poor provider adherence to guideline recommendations [2]. Attempts to improve outcomes through use of practice guidelines, quality improvement, or system improvement have not led to consistently positive effects on process of care measures or outcomes, and have been disappointing [3-5]. Organizational barriers, such as inadequate staff or support structure, are typically cited as a hindrance to guideline implementation [5].

To facilitate implementation, several models for delivery of care for patients with chronic disease have emerged. These include the "self-management" and "collaborative management" models, the "chronic care model," and the "disease management" approach [6-9]. Recent systematic reviews of organizational intervention studies based on the self-management, chronic care, and disease management models reveal small to moderate effects on process or outcome measures [6,10-13]. These results suggest that it may not be the content of the models, but rather, it may be that the specific way in which they are applied in organizations is critical.

One possible reason for the modest effects of organizational intervention outcomes is suggested by the recent application of complex adaptive system (CAS) theory to the organization of health care delivery [14-17]. A CAS is characterized by individuals who can learn, interconnect, self-organize, and co-evolve with their environment in non-linear dynamic ways [18,19]. These factors lead to patterns of relationships and interconnections in the system that influence performance of the system. Like musicians in an improvisational jazz band, individuals in a CAS learn from and react to each other and their environment, leading to constant shifts in the pattern of relationships or the interpretation of the music. The natural tendency of individuals to learn, form patterns of relationships, organize and evolve suggests that interventions that suppress these activities will lead to poorer outcomes than those that facilitate them.

CAS theory has been used to describe clinical settings, such as primary care clinics [15]. However, it also has implications for how we approach changing clinical systems [16,20], but the implementation literature is less developed than the descriptive literature. Using CAS theory as a framework to assist us in designing and implementing organizational interventions could potentially make these interventions more likely to achieve their aims.

Complexity science suggests that inconsistent outcomes may result from an implicit assumption of linear, mechanistic relationships between cause and effect in implementing organizational interventions. This linear viewpoint implies that a specific intervention should lead to consistent, reproducible results across clinical settings. In contrast, nonlinearity, self-organization, and co-evolution suggest that each clinical setting is unique, and that outcomes of interventions may be greatly affected by small situational differences. [18,19]. For example, clinical reminders may be very effective in prompting providers in one setting to perform recommended care, but may not be as effective in another because of very small differences in the settings (*i.e*., differences in the workflow of test ordering, ease of access of recommended care, patient population served, or provider beliefs).

The goal of this study is to build on the current literature related to using CAS to observe and understand clinical settings, to begin to examine the extent to which CAS theory is useful as a tool for designing and/or implementing organizational interventions. We hypothesize that interventions that leverage the characteristics of complex adaptive systems, intentionally or not, and regardless of the particular model of chronic care delivery on which the intervention was based, will consistently lead to improved outcomes over those that do not. To test this hypothesis, we conducted a systematic review of organizational interventions for patients with Type II diabetes and examine these interventions through the lens of complexity science.

## Methods

### Search strategy

We defined organizational interventions as those that explicitly attempt to affect or change organizational structures or processes to implement evidence-based practice. We searched Medline from 1989 through 29 December 2005, after developing a search strategy based on four components: 1) the strategy developed by the Effective Practice and Organization of Care (EPOC) Group of the Cochrane Collaboration and updated for a recent systematic review of Medicare-funded preventive services [21,22]; (2) additional search terms for types of organizational interventions not included in the EPOC search strategy, such as total quality improvement, PDSA (Plan-Do-Study-Act), and practice redesign; (3) additional search terms used by a recent systematic review of preventive and quality improvement strategies [23]; and (4) bibliographies and Medline indexing terms of relevant publications on organizational change and guideline implementation. To focus the search on diabetes, we added disease-specific MeSH and text word terms, ran a preliminary search, and reviewed 200 titles and abstracts (determined by saturation, until no further new terms were identified) for additional text word terms. The search terms are available in Additional File 1. We did not search the management literature, nor did we seek out unpublished data.

### Inclusion and exclusion criteria

Inclusion criteria were randomized, quasi-randomized, or controlled clinical trials published in English and conducted in economically developed countries as identified by the International Monetary Fund or the Organization for Economic Cooperation and Development [24]. We excluded non-English articles because non-English studies comprise only 1% of the EPOC registry. We excluded studies reporting only the following nonclinical outcomes: patient or provider knowledge; self-efficacy; satisfaction; or other attitudes and beliefs. We also excluded studies of: Type 1 or gestational diabetes; patients < 18 years old or patients younger and older than 18 but not reporting results separately for adults; work site health interventions; exercise rehabilitation or smoking cessation; and disease prevention or screening only.

Four investigators independently reviewed overlapping groups of differing halves of the citations' titles and abstracts generated by the full literature search to assess agreement regarding potentially eligible publications. Raw agreement was 94%. If eligibility was uncertain after review of the title and abstract, the full article was reviewed. Eligible studies were independently reviewed and jointly abstracted in detail by teams of two investigators. Disagreements were resolved by consensus of the group of investigators.

### Assessment of leveraging of characteristics of CAS

Eligible publications were independently evaluated by two raters with content expertise in complexity science to assess the extent to which the intervention utilized any of the following characteristics of a CAS: individuals' capacity/ability to learn; the interconnections between individuals; the ability of participants to self-organize; and the tendency of participants to co-evolve. These are representative of critical elements of CAS [18,19]. The definition of each characteristic used by reviewers is shown in Table 1. Each study was given a point for each of the characteristics present in the study design, for a possible lowest score of zero and highest score of four. Table 2 gives specific examples of interventions that met criteria for each score, with the characteristics they were felt to reflect. The raters were blinded to the outcomes of the studies. The kappa for these scores between reviewers was 0.78, with conflicts subsequently resolved by discussion.

**Table 1:** Characteristics of complex adaptive systems abstracted

Characteristic	Definition
Agents who learn	People can and will process information, as well as react to changes in information
Interconnections	Change in pattern of interactions, including non-verbal communication, among agents Introducing new agents into the system.
Self-organization	Order is created in a system without explicit hierarchical direction
Co-evolution	The system and the environment influence each other's development

**Table 2:** Examples of interventions utilizing characteristics of complex adaptive systems

Intervention	Characteristics Present	Score Given
1-page reminder of BP goals put on the front of the charts of all diabetics	None	0
Educational materials (articles, videotapes) sent to physicians at defined intervals	Learning	1
Decision – support system generated treatment recommendations based on current treatment and level of control. Patients seen monthly until controlled.	Interconnections Co-evolution	2
Pharmaco-evaluation and med review conducted at set intervals over 1 year. Emphasis on education, but tailored to progress of individual patients	Learning Interconnections Co-evolution	3
Usual visits replaced with group visits led by a physician and diabetes nurse educator, who were allowed to tailor the meeting frequency and content to the needs of the group. The goal of these visits was to improve compliance through education.	Learning Interconnections Self-Organization Co-evolution	4

### Assessment of reported outcomes

Because of the heterogeneity across study outcomes, we did not use effect size as the outcome variable. The most commonly used outcome was change in hemoglobin A1c, but this was used in only 14 studies of variable duration, four of which contained unit of allocation error, or mismatch between the unit of randomization and unit of analysis. Additionally, some studies compared change in hemoglobin A1c with baseline values, rather than with controls. To overcome this, a rating scale was used to assess the efficacy of the intervention. The outcomes of each study were assessed by two independent raters on a scale of 0 (no effect), 0.5 (mixed results), and 1 (intervention effective) based on the type (process versus outcome), number, and statistical significance of outcomes reported. The specific criteria used for each rating, along with specific examples of the reported outcomes for each rating, are shown in Table 3. Raters were blinded to study interventions, and one was different from the intervention raters. The kappa for these scores was 0.79, with conflicts resolved by discussion.

**Table 3:** Criteria used to classify outcomes of studies with organizational interventions

Outcome Score	Criteria	Example
0	No differences between control and intervention groups, or between intervention and baseline, on process or outcome measures	No difference in rate of medication changes between groups
0.5	Trends without significance Mixed outcomes (significant improvement in minority of measures) Significant improvement compared with baseline, but not with control	Significant improvement in hgb A1c at 6 and 12 months when compared with baseline, but not when compared with control group
1	Significant improvement: - all outcomes if ≤ 3 endpoints - majority of outcomes if > 3 endpoints	Significant improvement in number of patients at A1c goal, significant decrease in hospitalizations and emergency department visits

### Statistical analysis

Because of the small number of studies identified, we used Fisher's exact test to test the significance of the relationship between total number of characteristics of complex adaptive systems being leveraged by an organizational intervention and the strength of outcomes reported, as well as between each individual characteristic and the strength of outcomes. Because a mismatch between the unit of allocation and analysis may bias a study toward positive results, we divided studies into two groups based on whether a unit of analysis error was present. A second analysis using Fisher's exact test was performed including only those studies that did not contain a unit of analysis error. Finally, a third analysis using logistic regression was performed to weight studies based on both sample size and duration of intervention. All statistical analyses were performed using Stata 8.0 (College Station, Texas).

## Results

**Figure 1:**
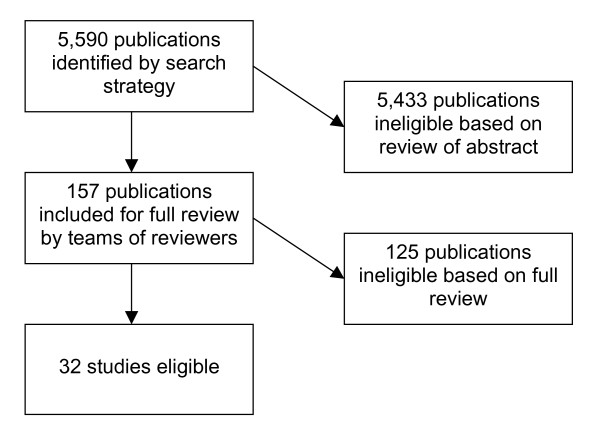
Flowchart of publication inclusion.

The search identified 5,590 publications; 157 were potentially eligible by review of title and abstract. After full review of these published studies, 32 met our eligibility criteria [25-56]. Figure 1 details the numbers of articles eligible and ineligible at each point. The interventions, outcomes, duration of intervention, presence of unit of allocation error, and extent to which interventions used characteristics of complex adaptive systems are summarized in Additional File 2. Half of studies reported significant improvement in most or all outcomes. Ten studies were felt to utilize self-organization; co-evolution was a component in twenty-four, learning in twenty-seven, and interconnections in thirty. Interventions that involved learning typically took the form of distribution of educational materials; interconnections were changed frequently through the addition of case managers or care coordinators. Relatively few (eight) allowed participants to self-organize.

The distribution of characteristics of complex adaptive systems utilized by a study intervention and the intervention efficacy for all studies is shown in Table 4. Only 50% of studies demonstrated effective results as reflected by a score of 1. All studies with an effectiveness score of 1 reported interventions whose CAS characteristic scores were at least 3, while the studies with lower effectiveness had lower CAS characteristic scores. The association between the number of CAS characteristics and effectiveness was statistically significant (p = 0.002). Analysis using logistic regression adjusting for sample size and duration of intervention remained significant. Nine studies had level of analysis error, most commonly because the unit of randomization was a clinic or physician, while the unit of analysis was the patient. The association between CAS characteristic scores and outcome effectiveness was also statistically significant when these nine studies were excluded (p = 0.004).

**Table 4:** Distribution of CAS and effectiveness of interventions

Total CAS Score	Rating of Intervention Effectiveness			Total No. Studies with each CAS Score
		
	0	0.5	1	
0	1	0	0	1
1	1	1	0	2
2	1	3	0	4
3	0	7	11	18
4	0	1	6	7
Total No. Studies at each Level of Effectiveness	3	12	17	32

p = 0.002

Of the four CAS characteristics assessed for each intervention, two were individually significantly associated with effectiveness. Interventions that affected interconnections in the organization or allowed participants to co-evolve were significantly associated with intervention effectiveness (p = 0.03 and 0.001, respectively). When studies with unit of analysis error were excluded, these associations remained significant (p = 0.05 and 0.003). The associations between interventions affecting participants' ability to learn or self-organize were not statistically significant (p = 0.06 and 0.58).

## Discussion

Consistent with prior literature, this systematic review found that many studies of organizational interventions do not improve process or outcome measures for patients with the chronic illness diabetes [3-5]. Only 50% of randomized or controlled clinical trials meeting criteria for this systematic review demonstrated significant improvement in most or all endpoints. In support of our hypothesis that implementation strategies consistent with CAS theory will be more likely to be effective, we found a significant relationship between the number of CAS characteristics utilized in an intervention and the intervention's effectiveness in improving process or outcome measures in patients with Type II diabetes. This was true in studies conducted across a diversity of clinical settings and reported outcomes. This finding potentially widens the scope of the application of CAS theory beyond the current literature that focuses on describing clinical settings as CAS [14,15], to build upon the idea that we can use CAS to both design and implement interventions [16,20] that are more likely to lead to improved performance.

Specifically, the finding that interventions incorporating features of CAS were more effective suggests that a dynamic approach to the design of organizational interventions to improve patient outcomes may be helpful. Rather than designing one-size-fits-all interventions that require adherence to a rigid, pre-set course of action, allowing participants to have input and control that reflect their local environment, as well as allowing them to adapt or deviate from a set plan as results evolve, may lead to more effective change. In short, we suggest that thinking about the intervention as a participatory, adaptive process rather than a set blueprint will lead to more effective interventions. These approaches currently exist; one example includes the Institute for Healthcare Improvement's Framework for Spread [57]. However, our finding that less than one-third of studies identified in our search allowed for self-organization suggests that their use is far from universal.

Two individual characteristics, co-evolution (ability of participants to modify practices based on forces internal and external to the clinical setting) and interconnections (changing the pattern of communication between participants) had the strongest relationship with intervention effect. It is difficult to interpret the lack of a relationship between other individual characteristics and strength of outcomes, as the number of studies included in this analysis was small, particularly after those with level of analysis error were excluded. However, the significant association between the overall number of characteristics of CAS and the intervention effectiveness, in the absence of a clear association for all individual characteristics, could imply that combinations of characteristics are more effective,*i.e*., the interventions should consider the holistic nature of practices. The number of studies was too small to allow for this analysis.

The association between interventions that influenced the interconnections between participants and intervention effectiveness may suggest that interventions that focus on the quality and pattern of relationships between participants in a clinical setting may be superior to those that focus on trying to change the behavior of a single participant. What sets this apart from other theories of organizational change is the scope of this approach to allow consideration of multiple aspects of the clinical setting, and the nonlinearity of the relationships between them. This inherent nonlinearity implies that inputs and outputs are unlikely to be proportional, particularly across clinical settings and over time, and suggests the need for the following specific approaches: involvement of more than one type of participant in both intervention design and implementation, use of more than one method of connecting agents in an organization, and continual reassessment of the effect of an intervention coupled with a willingness to make changes based on this reassessment. In fact, this may explain why multi-faceted organizational interventions may be more effective than those with a focus on a single strategy [58].

Several other limitations besides the small sample size deserve mention. All of the studies included related only to the chronic care of diabetic patients. Confirmation of these findings for patients with other chronic diseases is necessary. In addition, while we were able to address the potential bias inherent in studies with unit of allocation error, publication bias (*e.g*., positive studies being more likely to be published), or the possibility that studies with negative outcomes did not provide sufficient detail regarding interventions, could potentially impact these results. However, regarding the latter potential bias, the inter-rater consistency on characteristic ratings indicates that reported methods were sufficient to reliably assess the interventions. We did not identify studies in the management literature or seek out unpublished information or "grey literature." Our intention was to focus on studies in the peer-reviewed medial literature as the most simple, direct, and universally accepted approach to studying whether an association between interventions with features that reflect CAS and outcome effectiveness exists. If this association had not been found, our next step might have been to identify these additional sources.

The methodology of assigning scores retrospectively to both characteristics and outcomes is also potentially a limitation. Categorization based on a relatively small amount of data can be difficult, but we believe that the methods sections of the included studies contained enough information to give the reader at least a basic overview of all intervention components and thus a reliable assessment of utilization of CAS characteristics. It may also seem counterintuitive to apply a lens of nonlinearity to interventions conducted in the context of traditional randomized, quasi-randomized, or controlled clinical trials, and in fact it is near impossible to assess nonlinearity itself in the context of the format of a published interventional study. However, we do believe that enough information was present to make a determination of whether key characteristics of CAS were present in the intervention, and our inter-rater reliability supports this. We also believe that the characteristics of learning, interconnections, self-organization and co-evolution are the key concepts that are representative of CAS and its implications [18,19]; therefore, we do not believe it likely that other CAS experts would identify important elements whose basic meaning is not encompassed in these characteristics. It is possible that because all investigators come from a single institution or "school" of complexity, that the interpretation of a different group of investigators could be different, but we believe that because we focused on the key characteristics of CAS whose definitions are relatively well-established, this is less likely to have occurred. Finally, CAS characteristics may be inherent parts of several care delivery models; this is particularly true of learning and interconnections between individuals. However, the underlying CAS assumption of nonlinearity differentiates this perspective in a fundamental way.

While these results may be regarded as preliminary, they point to the use of complexity science as a framework for thinking about clinical settings that may allow us to better understand the inconsistencies in the health care organizational literature and to better design interventions that will lead to the greatest improvement in outcomes for our patients.

## Conclusion

Improved outcomes in Type II diabetes were significantly associated with organizational interventions that had characteristics of complex adaptive systems in their design. Those interventions incorporating a greater number of characteristics demonstrated the greatest improvement in diabetes-related outcomes. We observed a greater effect for interventions that promoted interconnections between, and co-evolution of, individuals.

These data may allow us to expand the framework of CAS from conceptualizing and studying clinical systems to encompass designing and implementing interventions that lead to improved patient outcomes. Specifically, interventions and implementation strategies that target multiple CAS characteristics may be most effective in improving health outcomes. Further research should address how best to translate the theoretical constructs of complex adaptive systems into interventions that improve the outcomes of chronically ill patients.

## Competing interests

The author(s) declare that they have no competing interests.

## Authors' contributions

LKL conceived this analysis using the database conceived by VL, PN, and JP, rated studies, performed preliminary statistical analysis, and drafted the manuscript. JP participated in the design of the study and helped to draft the manuscript. VL conceived the systematic review and database, rated studies, and helped to draft the manuscript. MP rated studies and helped to draft the manuscript. PN conceived the systematic review and database and helped to draft the manuscript. JC performed statistical analysis and helped to draft the manuscript. RMcD participated in the design of the study, provided theoretical expertise, and helped to draft the manuscript.

All authors have read and approved the final manuscript.

## Supplementary Material

Additional file 1 Search strategy for organizational interventions to improve outcomes of patients with Type II diabetes. Medline search strategy to identify studies of organizational interventions to improve outcomes for patients with Type II diabetes, as described in Leykum, et al, Organizational interventions employing principles of complexity science have improved outcomes for patients with Type II diabetes. Search completed December 2005. Click here for file

Additional file 2 Summary of included studies. Summary of eligible studies of organizational interventions on outcomes of patients with type 2 diabetes, as described in Leykum, et al, Organizational interventions employing principles of complexity science have improved outcomes for patients with Type II diabetes. Click here for file
