# Supplementary material for: Organizational interventions employing principles of complexity science have improved outcomes for patients with Type II diabetes
Source: Implement Sci. 2007 Aug 28;2:28. doi: 10.1186/1748-5908-2-28 (PMC2018702; doi:10.1186/1748-5908-2-28)
Supplement: Additional file 1 — Search strategy for organizational interventions to improve outcomes of patients with Type II diabetes. Medline search strategy to identify studies of organizational interventions to improve outcomes for patients with Type II diabetes, as described in Leykum, et al, Organizational interventions employing principles of complexity science have improved outcomes for patients with Type II diabetes. Search completed December 2005. [file 1748-5908-2-28-S1.doc]

**Additional File 1: Search Strategy to identify studies of organizational interventions to improve outcomes for patients with Type II diabetes, as described in Leykum, et al, Organizational interventions employing principles of complexity science have improved outcomes for patients with Type II diabetes. Search completed December 2005.**

|  | **Search History** | **Results** |
| --- | --- | --- |
| 1 | Quality of Health Care/ | 37901 |
| 2 | Quality Assurance, Health Care/ | 34221 |
| 3 | Quality Indicators, Health Care/ | 4535 |
| 4 | Quality Control/ | 28847 |
| 5 | (continuous adj5 quality adj improvement).tw. | 1502 |
| 6 | (institute adj2 healthcare adj improvement$).tw. | 113 |
| 7 | (total adj quality adj improvement$).tw. | 40 |
| 8 | (practice adj2 design$).tw. | 1084 |
| 9 | (practice adj2 redesign$).tw. | 29 |
| 10 | (practice adj2 engineer$).tw. | 62 |
| 11 | (practice adj2 reengineer$).tw. | 7 |
| 12 | (professional adj substitution$).tw. | 3 |
| 13 | (boundary adj encroachment).tw. | 2 |
| 14 | Program Evaluation/ | 26927 |
| 15 | PDSA.tw. | 49 |
| 16 | Practice Guidelines/ | 39779 |
| 17 | (practice adj guideline$).pt,tw. | 6432 |
| 18 | Guideline Adherence/ | 8623 |
| 19 | (guideline$ adj5 implement$).tw. | 2950 |
| 20 | (group adj visit$).tw. | 105 |
| 21 | Case Management/ | 5637 |
| 22 | Decision Making, Organizational/ | 8579 |
| 23 | Organizational Innovation/ | 15323 |
| 24 | Organizational Case Studies/ | 4518 |
| 25 | Organizational Culture/ | 7779 |
| 26 | models, organizational/ | 9630 |
| 27 | (organizational adj development$).tw. | 207 |
| 28 | (organizational adj change$).tw. | 911 |
| 29 | (organizational adj improvement$).tw. | 44 |
| 30 | (organizational adj intervention$).tw. | 58 |
| 31 | (organizational adj learning).tw. | 92 |
| 32 | (organizational adj2 objective$).tw. | 50 |
| 33 | (organizational adj2 outcome$).tw. | 129 |
| 34 | (organizational adj2 transformation$).tw. | 34 |
| 35 | organizational$.tw. | 16359 |
| 36 | "Outcome Assessment (Health Care)"/og [Organization & Administration] | 1097 |
| 37 | "Process Assessment (Health Care)"/og [Organization & Administration] | 168 |
| 38 | Outpatient Clinics, Hospital/og [Organization & Administration] | 1866 |
| 39 | Registries/ or registr$.tw. | 61108 |
| 40 | Clinical Pathways/ | 2782 |
| 41 | (shared adj care).tw. | 489 |
| 42 | or/1-41 | 283750 |
| 43 | Disease Management/ | 5254 |
| 44 | Patient Care Planning/ | 27305 |
| 45 | Patient-Centered Care/ | 4362 |
| 46 | Primary Health Care/ | 34103 |
| 47 | Progressive Patient Care/ | 1092 |
| 48 | Critical Pathways/ | 2782 |
| 49 | Delivery of Health Care, Integrated/ | 4992 |
| 50 | Health Services Accessibility/ | 28220 |
| 51 | Managed Care Programs/ | 21401 |
| 52 | Product Line Management/ | 1421 |
| 53 | Patient Care Team/ | 37625 |
| 54 | Behavior Control/ | 945 |
| 55 | Counseling/ | 20472 |
| 56 | Health Promotion/ | 29989 |
| 57 | Patient Compliance/ | 31658 |
| 58 | After-Hours Care/ | 308 |
| 59 | (coordination or coordinated or multifactorial or multi-factorial or multicomponent or multi-component or multidisciplinary or multi-disciplinary or interdisciplinary or inter-disciplinary or integrated or community-based or organized).tw. | 194565 |
| 60 | (care or approach or intervention or strategy or strategies or management or managing or center$ or clinic).tw. | 1632653 |
| 61 | 59 and 60 | 72726 |
| 62 | "Organization and Administration".sh. | 14063 |
| 63 | or/43-58,61-62 | 300845 |
| 64 | Total Quality Management/ | 9138 |
| 65 | Quality Control/ | 28847 |
| 66 | (tqm or tqi or cqu).tw. | 592 |
| 67 | (quality and (continuous or total)).tw. | 35944 |
| 68 | (total and (management or improvement)).tw. | 52171 |
| 69 | or/64-68 | 116317 |
| 70 | Education, Continuing/ | 5875 |
| 71 | (education and continuing and (medical or professional$ or nursing or physician$ or nurse$)).tw. | 7445 |
| 72 | (outreach and (visit or educational)).tw. | 510 |
| 73 | (academic and detailing).tw. | 197 |
| 74 | or/70-73 | 13549 |
| 75 | Diffusion of Innovation/ | 7109 |
| 76 | (diffusion and (innovation or technology)).tw. | 1021 |
| 77 | or/75-76 | 7811 |
| 78 | Medical Audit/ | 10428 |
| 79 | (audit or feedback or compliance or adherence or training).tw. | 261253 |
| 80 | (improvement$ or improving or improves or improve or guideline$ or practice$ or medical or provider$ or physician$ or nurse$ or clinician$).tw. | 1420484 |
| 81 | 79 and 80 | 96203 |
| 82 | Practice Guidelines/ | 39779 |
| 83 | (academic or visit$ or reminder$).tw. | 137326 |
| 84 | Reminder Systems/ | 1058 |
| 85 | ((financial or economic or physician$ or patient$) and incentive$).tw. | 4432 |
| 86 | Reimbursement Mechanisms/ | 7764 |
| 87 | or/78,81-86 | 278403 |
| 88 | Medical Informatics/ | 4000 |
| 89 | (computer or (decision and support)).tw. | 115418 |
| 90 | Telemedicine/ | 5504 |
| 91 | (telemedicine or telecommunication$ or web or modem or telephone$).tw. | 44132 |
| 92 | Internet/ | 23468 |
| 93 | Telephone/ | 6578 |
| 94 | or/88-93 | 178414 |
| 95 | or/63,69,74,77,81,87,94 | 803876 |
| 96 | outreach.tw. | 3874 |
| 97 | ((opinion or education$ or influential) adj leader$).tw. | 449 |
| 98 | facilitator$.tw. | 6728 |
| 99 | (academic adj detailing).tw. | 162 |
| 100 | (consensus adj conference).tw. | 2324 |
| 101 | *Guideline Adherence/ | 4091 |
| 102 | Practice Guidelines/ | 39779 |
| 103 | (practice adj guideline$).tw. | 6432 |
| 104 | (guideline$ adj2 (introduc$ or issu$ or impact or effect$ or disseminat$ or distribut$)).tw. | 2056 |
| 105 | ((effect$ or impact or evaluat$ or introduc$ or compar$) adj2 training program$).tw. | 421 |
| 106 | *Reminder Systems/ | 594 |
| 107 | reminder$.tw. | 3502 |
| 108 | (recall adj2 system$).tw. | 246 |
| 109 | (prompter$ or prompting).tw. | 2269 |
| 110 | algorithm$.tw. | 50661 |
| 111 | or/96-110 | 115542 |
| 112 | *Feedback/ or feedback.tw. | 44138 |
| 113 | (feedback adj (loop$ or control$ or regula$ or mechanism$ or inhib$ or system$ or circuit$ or sensory or visua or audio$ or auditory)).tw. | 16889 |
| 114 | 112 not 113 | 27249 |
| 115 | (chart adj review$).tw. | 10369 |
| 116 | ((effect$ or impact or records or chart$) adj2 audit).tw. | 562 |
| 117 | compliance.tw. | 49144 |
| 118 | marketing.tw. | 9905 |
| 119 | or/115-118 | 69539 |
| 120 | exp *Reimbursement Mechanisms/ | 12487 |
| 121 | (fee adj2 service).tw. | 2272 |
| 122 | *Capitation Fee/ | 1893 |
| 123 | "Deductibles and Coinsurance"/ | 1123 |
| 124 | (cost adj shar$).tw. | 620 |
| 125 | (copayment$ or (co adj payment$)).tw. | 608 |
| 126 | (prepay$ or prepaid or (prospective adj payment$)).tw. | 3559 |
| 127 | *Hospital Charges/ | 620 |
| 128 | formular$.tw. | 2280 |
| 129 | fundhold$.tw. | 359 |
| 130 | *Medicaid/ | 7700 |
| 131 | *Medicare/ | 13810 |
| 132 | Blue Cross/ | 1867 |
| 133 | or/120-132 | 39870 |
| 134 | *Nurse Clinicians/ | 4593 |
| 135 | *Nurse Practitioners/ | 8171 |
| 136 | *Nurses' Aides/ | 1939 |
| 137 | (nurse adj (rehabilitator$ or clinician$ or practitioner$)).tw. | 5626 |
| 138 | *Pharmacists/ | 4342 |
| 139 | (clinical adj pharmacist$).tw. | 706 |
| 140 | paramedic$.tw. | 3591 |
| 141 | psychologist$.tw. | 5881 |
| 142 | social workers.tw. | 3191 |
| 143 | dietician$.tw. | 469 |
| 144 | *Physician Assistants/ | 2110 |
| 145 | *Patient Care Team/ | 14504 |
| 146 | (team$ adj2 (care or treatment)).tw. | 5144 |
| 147 | (integrat$ adj (care or service$)).tw. | 1160 |
| 148 | (care adj (coordinat$ or program$ or continuity)).tw. | 5868 |
| 149 | *Case Management/ | 3519 |
| 150 | exp *Ambulatory Care Facilities/ | 18709 |
| 151 | *Ambulatory Care/ | 11622 |
| 152 | or/134-151 | 90207 |
| 153 | *Home Care Services/ | 15404 |
| 154 | *Nursing Homes/ | 15207 |
| 155 | *Office Visits/ | 1409 |
| 156 | *House Calls/ | 896 |
| 157 | *Day Care/ | 2517 |
| 158 | *Aftercare/ | 2177 |
| 159 | *Community Health Nursing/ | 12694 |
| 160 | (chang$ adj1 location$).tw. | 182 |
| 161 | domiciliary.tw. | 1722 |
| 162 | (home adj treat$).tw. | 887 |
| 163 | (day adj surgery).tw. | 1365 |
| 164 | *Medical Records/ | 13694 |
| 165 | *Medical Records, Computerized/ | 8445 |
| 166 | (information adj2 (management or system$)).tw. | 14941 |
| 167 | *Peer Review/ | 2580 |
| 168 | *Utilization Review/ | 2324 |
| 169 | *Health Services Misuse/ | 1550 |
| 170 | or/153-169 | 91916 |
| 171 | *Physician's Practice Patterns/ | 12844 |
| 172 | *Quality Assurance, Health Care/ | 19315 |
| 173 | *"Process Assessment (Health Care)"/ | 873 |
| 174 | *ProGram Evaluation/ | 3946 |
| 175 | *Length of Stay/ | 4683 |
| 176 | (early adj discharge).tw. | 1300 |
| 177 | (discharge adj planning).tw. | 1525 |
| 178 | offset.tw. | 10028 |
| 179 | triage.tw. | 4430 |
| 180 | exp *"Referral and Consultation"/ | 15803 |
| 181 | *Drug Therapy, Computer Assisted/ | 652 |
| 182 | (near adj patient adj testing).tw. | 139 |
| 183 | *Medical History Taking/ | 3322 |
| 184 | *Telephone/ | 3136 |
| 185 | *Physician-Patient Relations/ | 20077 |
| 186 | *Health Maintenance Organizations/ | 9057 |
| 187 | *Managed Care Programs/ | 14692 |
| 188 | (hospital$ adj merg$).tw. | 273 |
| 189 | or/171-188 | 120897 |
| 190 | ((standard or usual or routine or regular or traditional or conventional or pattern) adj care).tw. | 5542 |
| 191 | (program$ adj (reduc$ or increas$ or decreas$ or chang$ or improv$ or modify$ or monitor$ or care)).tw. | 2525 |
| 192 | (program adj (health or care or intervention)).tw. | 245 |
| 193 | (((effect$ or impact or evauat$ or introduc$ or compar$) adj (treatment or care or screening or prevent$)) and program$).tw. | 2040 |
| 194 | (computer$ adj (dosage or dosing or diagnosis or therapy or decision$)).tw. | 554 |
| 195 | (protocol$ adj (introduc$ or impact or effect$ or implement$ or computers$)).tw. | 222 |
| 196 | ((effect$ or impact or introduc$) adj (legislation or regulation$ or policy)).tw. | 420 |
| 197 | Community Health Services/ | 21259 |
| 198 | Research/og | 1882 |
| 199 | "Outcome and Process Assessment (Health Care)"/ | 15557 |
| 200 | or/190-199 | 49599 |
| 201 | or/111,114,119,133,152,170,189,200 | 541639 |
| 202 | or/42,95,201 | 1237863 |
| 203 | Diabetes Mellitus/ | 67437 |
| 204 | Diabetes Mellitus, Non-Insulin-Dependent/ | 44180 |
| 205 | Diabetes Mellitus, Type II/ | 44180 |
| 206 | (diabetes and (type adj II)).tw. | 5173 |
| 207 | (diabetes and type 2).tw. | 24252 |
| 208 | NIDDM.tw. | 6512 |
| 209 | (diabet$ or glycemic or glycaemic).tw. | 239318 |
| 210 | exp Diabetic Angiopathies/ | 27187 |
| 211 | Diabetic Nephropathies/ | 13632 |
| 212 | Diabetic Neuropathies/ | 9290 |
| 213 | or/203-212 | 265661 |
| 214 | 213 not Diabetes, Gestational/ | 263137 |
| 215 | 202 and 214 | 26861 |
| 216 | limit 215 to yr=1989-2004 | 17769 |
| 217 | limit 216 to english language | 15500 |
| 218 | limit 217 to human | 14837 |
| 219 | 218 not (child or infant$ or neonat$ or adolesc$ or pediatr$).sh,pt,tw,jn. | 12023 |
| 220 | 219 not (letter or editorial or comment).sh,pt,tw. | 10499 |
| 221 | 220 not Case Report.sh,pt,tw. | 10463 |
| 222 | Randomized Controlled Trials/ | 49574 |
| 223 | Randomized Controlled Trial.pt. | 238087 |
| 224 | exp Controlled Clinical Trials/ | 52643 |
| 225 | Controlled Clinical Trial.pt. | 75309 |
| 226 | Random Allocation/ | 58314 |
| 227 | Double-Blind Method/ | 92083 |
| 228 | Single-Blind Method/ | 11082 |
| 229 | or/222-228 | 406274 |
| 230 | 221 and 229 | 1109 |
| 231 | exp Clinical Trials/ | 193436 |
| 232 | Clinical Trial.pt. | 437587 |
| 233 | (clin$ adj trial$).tw. | 108608 |
| 234 | ((singl$ or doubl$ or trebl$ or tripl$) adj25 (blind$ or mask)).tw. | 88906 |
| 235 | Placebos/ | 26294 |
| 236 | placebo$.tw. | 103373 |
| 237 | random$.tw. | 376892 |
| 238 | exp Research Design/ | 221844 |
| 239 | or/231-238 | 895295 |
| 240 | 221 and 239 | 2342 |
| 241 | Comparative Study/ | 0 |
| 242 | exp Evaluation Studies/ | 604397 |
| 243 | Follow-Up Studies/ | 340983 |
| 244 | Prospective Studies/ | 223769 |
| 245 | Multicenter Studies/ | 10916 |
| 246 | (control$ or prospectiv$ or volunteer$).tw. | 1799876 |
| 247 | or/241-246 | 2598025 |
| 248 | 221 and 247 | 4777 |
| 249 | (time adj series).tw. | 6470 |
| 250 | ((pre adj test) or pretest or (post adj test) or posttest).tw. | 7812 |
| 251 | (case adj (study or studies)).tw. | 25845 |
| 252 | or/249-251 | 39972 |
| 253 | 221 and 252 | 156 |
| 254 | or/230,240 | 2348 |
| 255 | or/248,253 | 4857 |
| 256 | 254 and 200410$.em. | 27 |
| 257 | 254 and 200411$.em. | 30 |
| 258 | 254 and 200412$.em. | 34 |
| 259 | 254 and 200501$.em. | 31 |
| 260 | 254 and 200502$.em. | 38 |
| 261 | 254 and 200503$.em. | 28 |
| 262 | 254 and 200504$.em. | 13 |
| 263 | 254 and 200505$.em. | 13 |
| 264 | 254 and 200506$.em. | 6 |
| 265 | 254 and 200507$.em. | 2 |
| 266 | 254 and 200508$.em. | 0 |
| 267 | 254 and 200509$.em. | 2 |
| 268 | or/256-267 | 224 |
