# Supplementary material for: Organizational interventions employing principles of complexity science have improved outcomes for patients with Type II diabetes
Source: Implement Sci. 2007 Aug 28;2:28. doi: 10.1186/1748-5908-2-28 (PMC2018702; doi:10.1186/1748-5908-2-28)
Supplement: Additional file 2 — Summary of included studies. Summary of eligible studies of organizational interventions on outcomes of patients with type 2 diabetes, as described in Leykum, et al, Organizational interventions employing principles of complexity science have improved outcomes for patients with Type II diabetes. [file 1748-5908-2-28-S2.doc]

**Additional File 2: Summary of eligible studies of organizational interventions on outcomes of patients with type 2 diabetes, as described in Leykum, et al, Organizational interventions employing principles of complexity science have improved outcomes for patients with Type II diabetes.**

| Author | Sample Size | Intervention | Outcome  Measures | Follow-up | Unit  of Analysis Error | CAS  Leveraged |
| --- | --- | --- | --- | --- | --- | --- |
| Denver,  EA (25) | 120 | Nurse-led clinic  Patient education | Systolic BP, target BP  Medication changes,  CHD risk scores | 6 mos | No | L, IC, SO, CoE |
| Frijling, BD (26) | 1449 | Physician feedback reports  Outreach visits to physicians | Process measures:  Foot, eye exams | 21 mos | Yes | L, IC, SO, CoE |
| Gary, TL (27) | 186 | Nurse Case Managers  Case Workers, or both | Hemoglobin a1c  Cholesterol, BP | 24 mos | No | L, IC, CoE |
| Glasgow, RE (28) | 320 | Health, diet counselor  Telephone outreach  Community resources | Hemoglobin A1c  Lipid profile  Psychosocial outcomes | 12 mos | No | L, IC, CoE |
| Hirsh, IB (29) | 185 | Computerized compliance feedback  Physician didactic training | Hemoglobin A1c (% change)  Weight, BP, costs | 14 mos | Yes | L, IC, CoE |
| Kim, HS (30) | 50 | Care booklet and log  Nurse phone intervention  Dietician review | Hemoglobin A1c  Diet, exercise, glucose monitoring adherence | 12 weeks | No | L, IC, CoE |
| Miller, CD (31) | 597 | Rapid a1c at time of visit  Education | Treatment intensification  Hemoglobin A1c | 4-8 mos | No | L, IC, CoE |
| Oh, JA (32) | 50 | Care booklet, logs  Phone calls  Dietician recommendations | Hemoglobin A1c  Blood glucose  BMI | 12 weeks | No | L, IC, SO, CoE |
| Sanders, K (33) | 320 | Colored chart reminders | Medication changes | unclear | Yes | IC |
| Stroebel, R (34) | 1083 | Searchable patient registry  Team time  Letters to patients | % pts with hgb A1c >8  Lipids ordered  BP | 6 mos | No | L, IC, SO |
| Greoneveld,Y (35) | 288 | Visits with nurse educator and dietician for 1 year | Hemoglobin A1c  Specialist referral | 12 mos | Yes | L, IC, CoE |
| McDermott, RA (36) | 678 | Outreach groups  Recall system | Process measures: BP, weight check, eye, foot care | 12 mos | No | L, IC, SO, CoE |
| Piette, JD (37) | 292 | Telemedicine system  Nurse educators in VA | Hemoglobin A1c  Process measures: foot exams, cholesterol, glucose monitoring | 12 mos | No | L, IC, CoE |
| Piette, JD (38) | 280 | Telemedicine system  Nurse educators in community health centers | Hemoglobin A1c  Process measures: foot exams, cholesterol, glucose monitoring | 12 mos | No | L, IC, CoE |
| Pritchard, DA (39) | 17 | Dieticians  PCP focus on diet | Weight  Blood pressure | 12 mos | No | L, IC, CoE |
| Wagner, EI (40) | 35 | Small group visits  Counseling | Hemoglobin A1c  Preventive care  ER visits, bed days | 24 mos | Yes | L, IC, SO, CoE |
| Walker EA (41) | 600 | PCP workshops  Project liaisons | Process measures: hgb A1c measurement, foot exams | 12 mos | Yes | L, IC |
| Cagliero, E (42) | 89 | Hgb a1c at time of visit | Hemoglobin A1c  Resource utilization | 12 mos | No | IC, CoE |
| Vaughan, NJ (43) | 218 | Decision support system | # pts with normal A1c | 16 mos | Yes | IC, CoE |
| Coffey, E (44) | 96 | Managed care vs. Fee for service systems | Medications, non-drug treatment  Pt income spent | 12 mos | No | - |
| Litzelman, DK (45) | 396 | MD guidelines re: foot care  Pt ed, Behavior contract  Phone/mail reminders | Foot lesions  Foot care behavior | 12 mos | Yes | L, IC |
| Newcomb, PA (46) | 1082 | Mobile eye exams  Education, phone contact | Visual impairment  Ophthalmology visits | 7-9 years | No | L, IC` |
| Clancy, DE (47) | 120 | Group visits | Process indicators  Hemoglobin A1c  Cholesterol | 6 mos | No | L, IC, SO, CoE |
| McClellan, W (48) | 123 | Physician-based QI project | Process indicators: A1c, urine testing, eye exams | 6 mos | No | L |
| Taylor, CT (49) | 69 | Pharmacoevaluation  Education | # pts with A1c at goal, BP  Hospital utilization  Quality of life | 12 mos | No | L, IC, SO, CoE |
| Tsuyuki, RT (50) | 675 | Visits with pharmacist | Composite lipid panel or increase in lipid medication | 16 weeks | No | L, IC, SO, CoE |
| Basch, CE (51) | 280 | Eye education, phone calls | Receipt of eye exam | 6 mos | No | L, IC, CoE |
| McCabe, C (52) | 2001 | Foot-screening protocol | Ulcers, amputations | 24 mos | No | L, IC, CoE |
| Lobach, DF (53) | 1265 | Computer-assisted management protocol | Guideline adherence,  Compliance, time spent | 6 mos | No | IC, SO, CoE |
| Shultz, EK (54) | 20 | Modem transfer of blood glucose measurements | Hemoglobin A1c (exact change not quantified) | 15 mos | No | L, IC, CoE |
| Smith, S (55) | 183 | PCP education / protocols  Community nurse specialist | Hemoglobin A1c, BP, BMI  Satisfaction, Well-being | 18 mos | Yes | L, IC, CoE |
| Rothman, RL (56) | 217 | Pharmacists  Diabetes care coordinator | Hemoglobin A1c  BP, cholesterol  Aspirin use | 12 mos | No | L, IC, CoE |

L = Learning, IC = Interconnections, SO = Self-organization, CoE = Co-evolution
